# Neuronopathic Gaucher disease: Beyond lysosomal dysfunction

**DOI:** 10.3389/fnmol.2022.934820

**Published:** 2022-08-03

**Authors:** Nohela B. Arévalo, Cristian M. Lamaizon, Viviana A. Cavieres, Patricia V. Burgos, Alejandra R. Álvarez, María J. Yañez, Silvana Zanlungo

**Affiliations:** ^1^Department of Gastroenterology, Faculty of Medicine, Pontificia Universidad Católica de Chile, Santiago, Chile; ^2^Department of Cell and Molecular Biology, Biological Sciences Faculty, Pontificia Universidad Católica de Chile, Santiago, Chile; ^3^Departamento de Biología Celular y Molecular, Facultad de Ciencias Biológicas, Millennium Institute on Immunology and Immunotherapy, Pontificia Universidad Católica, Santiago, Chile; ^4^Facultad de Medicina y Ciencia, Centro de Biología Celular y Biomedicina (CEBICEM), Universidad San Sebastián, Santiago, Chile; ^5^Facultad de Ciencias Biológicas, Centro de Envejecimiento y Regeneración (CARE-UC), Pontificia Universidad Católica, Santiago, Chile; ^6^Centro Ciencia & Vida, Fundación Ciencia & Vida, Santiago, Chile; ^7^Faculty of Medicine and Science, School of Medical Technology, Universidad San Sebastian, Concepción, Chile

**Keywords:** lysosomal storage disorders (LSD), Neuronopathic Gaucher disease (nGD), Parkinson's disease (PD), lysosome, autophagy, endoplasmic reticulum, mitochondria, Golgi apparatus

## Abstract

Gaucher disease (GD) is an inherited disorder caused by recessive mutations in the *GBA1* gene that encodes the lysosomal enzyme β-glucocerebrosidase (β-GC). β-GC hydrolyzes glucosylceramide (GluCer) into glucose and ceramide in the lysosome, and the loss of its activity leads to GluCer accumulation in different tissues. In severe cases, enzymatic deficiency triggers inflammation, organomegaly, bone disease, and neurodegeneration. Neuronopathic Gaucher disease (nGD) encompasses two different forms of the disease, characterized by chronic or acute damage to the central nervous system (CNS). The cellular and molecular studies that uncover the pathological mechanisms of nGD mainly focus on lysosomal dysfunction since the lysosome is the key organelle affected in GD. However, new studies show alterations in other organelles that contribute to nGD pathology. For instance, abnormal accumulation of GluCer in lysosomes due to the loss of β-GC activity leads to excessive calcium release from the endoplasmic reticulum (ER), activating the ER-associated degradation pathway and the unfolded protein response. Recent evidence indicates mitophagy is altered in nGD, resulting in the accumulation of dysfunctional mitochondria, a critical factor in disease progression. Additionally, nGD patients present alterations in mitochondrial morphology, membrane potential, ATP production, and increased reactive oxygen species (ROS) levels. Little is known about potential dysfunction in other organelles of the secretory pathway, such as the Golgi apparatus and exosomes. This review focuses on collecting evidence regarding organelle dysfunction beyond lysosomes in nGD. We briefly describe cellular and animal models and signaling pathways relevant to uncovering the pathological mechanisms and new therapeutic targets in GD.

## Introduction

Gaucher disease (GD) is the most common lysosomal storage disorder (LSD), with a prevalence of 1.75:100,000 people worldwide (Nalysnyk et al., 2017; Institute of Gaucher Disease, 2020) and a higher incidence in populations with specific genetic backgrounds, such as Ashkenazi Jews (1:800) (Rosenbloom and Weinreb, 2013; Stirnemann et al., 2017).

GD pathology results mainly from recessive mutations in the *GBA1* gene. This gene encodes the lysosomal enzyme β-glucocerebrosidase (β-GC) that hydrolyzes glucosylceramide (GluCer) into glucose and ceramide (Brady et al., 1966; Rosenbloom and Weinreb, 2013). There are more than 300 mutations that lead to a deficiency or insufficient activity of β-GC, which induces GluCer accumulation in the lysosomes (Hruska et al., 2008; Database The Human Genome, 2020). Macrophages are the most commonly affected cells in GD, called Gaucher cells (Lee, 1968). These cells are characterized by nuclear displacement, lysosomal swelling, and cytoplasm with a crumpled paper appearance (Lee, 1968). However, GluCer accumulation is also observed in macrophages from the reticuloendothelial system (such as Kupffer cells) (Brady, 1997; Simpson et al., 2014; Degnan et al., 2019) and neurons (Farfel-Becker et al., 2014).

There is also an abnormal accumulation of secondary lipids in addition to GluCer. For example, there is an increase in the deacetylated form of GluCer, Glucosylsphingosine (GluSph), in patient's cerebral and cerebellar cortices and GD animal models (Nilsson and Svennerholm, 1982; Orvisky et al., 2002; Dekker et al., 2011; Stiles et al., 2021). This buildup can start as early as conception and is linked to genetic severity (Orvisky et al., 2002). The GluSph toxic buildup is thought to cause neuronal death in GD2 *via* neurophagy (Nilsson and Svennerholm, 1982). For example, a study on LA-N-2 cells found that increasing GluSph treatment distorts neuronal morphology, declining neurite growth and acetylcholine production (Schueler et al., 2003). Similarly, it has been shown that GluSph can cause abnormal cell consequences such as inflammation, α-synuclein (α-syn) accumulation, and altered cell viability (Revel-Vilk et al., 2020). Although the exact mechanism by which GluSph contributes to GD2 disease is unknown, it is thought that GluSph blocks β-GC activity, establishing a negative feedback mechanism (Schueler et al., 2003; Revel-Vilk et al., 2020). In addition, cholesterol buildup has been linked to GD (Puri et al., 1999; Salvioli et al., 2005; Ron and Horowitz, 2008; Yañez et al., 2020). Fibroblasts from GD patients treated with inhibitors of cholesterol production reduce endoplasmic reticulum-associated responses and improve β-GC stability, maturation, and localization (Ron and Horowitz, 2008). Furthermore, cholesterol accumulation disrupts membrane traffic in the endocytic pathway in LSD, implying that secondary lipid accumulation contributes to GD pathogenesis. The abnormal accumulation of lipids in lysosomes results in clinical manifestations such as splenomegaly, hepatomegaly, bone disease, hematological alterations, and, in severe cases, neurodegeneration (Stirnemann et al., 2017). Indeed, the central nervous system (CNS) involvement has been a key determinant of GD classification. GD type 1 (GD1, OMIM #230800), the most prevalent form covering 90–95% of GD cases, is characterized by systemic manifestations (Stirnemann et al., 2017). GD type 2 (GD2, OMIM #230800) and GD type 3 (GD3, OMIM #230900) show CNS damage and are known as neuronopathic GD (hereafter nGD, unless its distinction is stated) (Stirnemann et al., 2017).

GD2 is the severe and progressive neuronopathic form of GD that manifest prenatally or during the first 9 months of life (Mignot et al., 2006). Although it contributes to <5% of cases, this form has a poor prognosis with non-effective treatment, and usually, children die before 2 years of age, with an average life expectancy of 11.7 months (Mignot et al., 2006). The low residual β-GC activity (10–15%) gives rise to the GD2 triad that encompasses neck rigidity, swallowing disorders, and oculomotor paralysis, along with systemic manifestations of the GD1 form (Weiss et al., 2015). Furthermore, GD2 could be accompanied by hydrops fetalis, in which fluids accumulate in different regions; however, these cases are perinatal lethal (Weiss et al., 2015). Furthermore, due to altered ceramide/glucosylceramide ratios in the skin, children present congenital ichthyosis. Organomegaly can be detected at birth (Weiss et al., 2015). This enlargement can be severe and affect feeding and breathing (Weiss et al., 2015).

Nevertheless, the prominent characteristic is the neurological decline due to a severe and rapidly progressive brainstem degeneration in the foreground (Mignot et al., 2006; Weiss et al., 2015). Patients frequently present the GD2 triad with arthrogryposis and microcephaly hypokinesia (Mignot et al., 2006; Weiss et al., 2015). The mechanism that leads to CNS dysfunction in GD2 is unknown; however, it is well-known that GluCer accumulation in neurons exceeds a threshold after which the inflammatory process and cell death begin (Farfel-Becker et al., 2014). This phenotype is characterized by microglial activation, astrocytosis, and neuronal death in layers III and V of the cortex and the CA2-CA4 hippocampal regions (Wong et al., 2004; Farfel-Becker et al., 2011, 2014). GD2 models present synaptic dysfunction, reduced synaptic size (Ginns et al., 2014), attenuated long-term potentiation (Sun et al., 2010), and alterations in synaptic proteins (Rocha et al., 2015).

GD3 (OMIM #231000) corresponds to a chronic neuronopathic form and encompasses 5–33% of GD cases (Stirnemann et al., 2017). The first manifestations start before 2 years of age (Tylki-Szymańska et al., 2010) but have a better prognosis than GD2 since patients can live over three decades (Schwartz et al., 2018). Oculomotor symptoms, cognitive deterioration, and seizures are the primary manifestation of CNS dysfunction; these symptoms occur along with systemic alterations.

Studying nGD remains a challenge to researchers since there is no clear relationship between the genotype and phenotype (Goker-Alpan et al., 2003). Although there are common mutations for specific subtypes, the same modifications could give rise to differences in severity, especially if there is CNS involvement (Goker-Alpan et al., 2003). Moreover, *GBA1* mutations are one of the main risk factors for developing Parkinson's disease (PD) (Aflaki et al., 2017). It is still a matter of debate whether GD patients share pathophysiological characteristics, such as α-syn aggregation (Aflaki et al., 2017), with PD patients since some authors have shown that α-syn inclusion is present in GD patients with parkinsonism (Wong et al., 2004), while others did not detect α-syn inclusions in GD2 brains (Berger-Sieczkowski et al., 2016). In this regard, the relationship between GD and PD is an emerging and intriguing topic.

Cellular and molecular studies of nGD focus mainly on lysosomal dysfunction. However, several studies have reported that alterations in other cell organelles can also contribute to the pathology. For example, excessive calcium release from the endoplasmic reticulum (ER) leads to overactivation of ER-associated degradation (ERAD) and the unfolded protein response (UPR). Likewise, alterations in mitochondrial membrane potential, adenosine triphosphate (ATP) levels, and reactive oxygen species (ROS) have been observed. In this review, we focused on collecting and discussing the evidence regarding different dysfunctional organelles in GD. We review the knowledge considering the nGD model's main limitations and how this affects the interpretation of the results to uncover pathological mechanisms in nGD.

## An overview of animal and cellular models of GD

The interpretation of results emerging from various cellular and animal models presenting a broad spectrum of pathological changes and differences is a major challenge in studying nGD. The first models of GD pathology started with the discovery of the selective and irreversible β-GC inhibitor conduritol-β-epoxide (CβE). Kanfer et al. used intraperitoneal and subcutaneous doses of CβE to treat mice. These animals present β-GC activity lower than 10%, showing the accumulation of GluCer in the brain, liver, and spleen (Kanfer et al., 1982). Variations in the treatment duration (Vardi et al., 2016), starting age of treatment (Marshall et al., 2002; Xu et al., 2008; Vardi et al., 2016), and genetic background were tested to improve GD characteristics (Klein et al., 2016; Vardi et al., 2016). Different genetic backgrounds treated with CβE presented distinct disease severity and progression (Klein et al., 2016). This model bias can complicate the interpretation of the results.

Neuronal lines and primary cultures treated with CβE have been used to generate GD cellular models. These pharmacological models are widely used since they are easy and fast to create and exhibit the main features of GD. Monocytes from GD patients have been used as a GD genetic model; however, their use is limited because of their low growth capacity (Aflaki et al., 2014; Bettman et al., 2015). Nevertheless, using the CRISPR/CAS9 technique, *GBA1* silencing was triggered in the monocyte cell line THP-1 and glioblastoma U87 (Pavan et al., 2020). Both lines showed β-GC activation of <1% accompanied by accumulation of GluCer (Pavan et al., 2020). In addition, particular disease characteristics were reproduced, such as enzyme retention in the endoplasmic reticulum (ER) and activation of the response to misfolded proteins (UPR) (Pavan et al., 2020). Skin fibroblasts are also used as a genetic model of GD; however, these are not the primary cell type affected in nGD (Danes and Bearn, 1968). Nevertheless, it has been possible to obtain different cell types affected by the disease, such as osteoclasts (Panicker et al., 2018), macrophages (Panicker et al., 2012, 2014; Aflaki et al., 2014; Messelodi et al., 2021), and neurons (Schöndorf et al., 2014) from induced pluripotent stem cells (iPSCs) derived from GD fibroblasts (Santos and Tiscornia, 2017). Furthermore, the immortalization of cortical neurons from *GBA1*^−/−^ mouse embryos provides a new tool for studying nGD (Westbroek et al., 2016). These GD neurons show a severe loss of more than 90% β-GC activity, GluCer accumulation, enlarged lysosomes, and altered calcium homeostasis compared with *GBA1*^+/+^ controls (Westbroek et al., 2016). These cell models are summarized in Table 1.

**Table 1:** Cellular models designed for GD study.

**Cell model/Origine**	**Methodology**	**β-GC activity**	**Lipid accumulation**	**Altered signal pathways and cellular process**	**References**
iPSC-derived midbrain dopaminergic neurons (Fibroblasts from GD1 and GD3 patients)	Skin fibroblasts reprogramming	<30%	GluCer	↑α-syn ↓ GBA2, β-galactosidase activity ↑ Number and size of LAMP1^+^ particles ↑ Number of LC3^+^ particles and LC3 II levels ↓ Autophagic flux ↓ Autophagosome-lysosome fusion Autophagy ↑ Basal Ca^2+^ levels and RyR-mediated calcium release	
					Schöndorf et al., 2014
iPSC-neuronal cells (Fibroblasts from GD1, GD2, and GD3 patients)	Skin fibroblasts reprogramming	<25%	GluCer	↓ LAMP1^+^ particles and altered clustering ↓ Lysosome number ↓ Cathepsin B, D, hexosaminidase A, and glucosamine (N-acetyl)-6-sulfatase transcripts ↑ LC3 II and p62/SQSTM1 levels ↓ Authofagosome clearance ↓ TFEB levels, ↑ TFEB nuclear localization ↑ mTOR activity	
					Panicker et al., 2012; Awad et al., 2015, 2017; Brown et al., 2019
Gba^−/−^ Immortalized neurons (*Gba1* Knockout mice)	EF1α-SV40T lentivirus immortalization	3%	GluCer GluSph	↑ Lysosome size and number ↓ ATP-dependent Ca^2+^ response ↓ Mitochondria basal and maximal respiration ↓ ATP production ↓ Mitochondrial Membrane Potential ↑ VDAC, Tom20, LC3 II levels ↑ mTOR activity ↑ TFEB levels	
					Westbroek et al., 2016; Peng et al., 2021
U87^GBA1−/−^ (Human glial cells)	CRISPR-Cas9	<1%	GluCer GluSph	↑ BiP and Chop transcripts ↑β-GC ERAD degradation ↑ Interleukin-1β ↑α-syn ↑ % Apoptotic cells	
					Pavan et al., 2020
Fibroblasts^*^ (Fibroblasts from GD1, GD2, and GD3 patients)	–	<10%	GluCer GluSph	↑*Itch* transcripts ↑ Bip, Chop, Xbp1 transcripts ↑ eIF2α phosphorylation ↑β-GC-calnexin colocalization ↑β-GC-ERAD degradation ↓ Mitochondrial complex I, II, III, and II + III activities ↓ CoQ content ↓ Mitochondrial membrane potential ↓ ATP levels ↑ ROS production	
					Mu et al., 2008; Wei et al., 2008; Maor et al., 2013b; de la Mata et al., 2015; Yañez et al., 2020, 2021
				↑ Lysosome number ↑ LC3 II/LC3 I ratio ↑ BECLIN1 levels ↑ Autophagosome formation ↑ Cathepsin D levels ↑ c-Abl and RIPK3 levels ↓ Autophagy	
iPSC-derived Osteoblasts (Fibroblasts from GD1, GD2, and GD3 patients)	Skin fibroblasts reprogramming	<25%	GluCer	↓ ALP, Col1, Runx2 transcripts ↓ ALP activity ↓ Activated β-catenin levels ↓ pGSK3β(S9) levels ↓ LAMP1 levels ↓ Cathepsin B, L, D, and ALS activity ↓ Ca^2+^-dependent exocytosis and plasma membrane repair	
					Panicker et al., 2018
Primary macrophages (Human monocytes from GD1 patients)	–	11.2–21%	GluCer GluSph	↓ ROS production for bacteria phagocytosis ↓ CCL5, CXCR4, MCP2 chemokines	
					Aflaki et al., 2014
iPSC-derived Macrophages (Fibroblats from GD1 patients)	Fibroblast reprogramming	3–20%	GluCer GluSph	↓ ROS production for bacteria phagocytosis ↓ Chemotaxis	
iPSC-macrophages (Fibroblasts from GD1, GD2, and GD3 patients)	Skin fibroblasts reprogramming	<5%	GluCer	↓ Red blood cell (RBC) clearance ↑ TNFα, IL-6, and IL-1β levels ↑ Chitotriosidase activity	
					Panicker et al., 2012, 2014
iPSC-Derived Monocyte/Macrophage (PBMCs from GD1 patient)	PBMCs reprogramming	<10%	GluCer	↓ Growth capacity ↑ RIPK3, MLKL transcripts	
					Messelodi et al., 2021
THP-1^GBA1−/−^ (Human monocytes)	CRISPR-Cas9	<26%	GluCer GluSph	NA	
					Pavan et al., 2020

* The particular characteristics of GD fibroblasts depend on the specific mutations (N370S, L444P, D409H, RecNil, and others). This table provides an overview of what has been observed in fibroblasts from patients with these mutations (Mu et al., 2008; Wei et al., 2008; Panicker et al., 2012, 2014, 2018; Maor et al., 2013b; Aflaki et al., 2014; Schöndorf et al., 2014; Awad et al., 2015, 2017; de la Mata et al., 2015; Brown et al., 2019; Yañez et al., 2020, 2021). NA, Not described; ↑, increased; ↓, decreased.

The first *GBA1* knock-out mice generated showed <4% β-GC. This animal model showed GluCer accumulation mimicking nGD characteristics; nevertheless, these mice died 1 day after birth (Table 1) (Tybulewicz et al., 1992). Later, two mice with *GBA1* point mutations were generated by inserting the recombinant mutation RecNcil or the L444P mutation (Liu et al., 1998) (Table 1). The L444P model was further improved by introducing a point mutation in the gene that encodes glucosylceramide synthase to attenuate GluCer synthesis (Mizukami et al., 2002). These mice displayed lower β-GC activity levels but did not present Gaucher cell characteristics (Table 1) (Mizukami et al., 2002). Xu et al. also generated mice models with *GBA1* homozygous point mutations or heterozygous point mutations/null alleles (N370S, V394L, D409H, and D409V). Some of these animals survived longer than 60 weeks and presented Gaucher cells; however, none developed CNS involvement, confirming the defects in the brain are caused by GluCer accumulation (these and other models are summarized in Table 1) (Xu et al., 2003).

Several strategies have been used to eradicate β-GC activity in tissues, such as hematopoietic or mesenchymal cells (Enquist et al., 2006; Sinclair et al., 2007; Mistry et al., 2010). One such strategy was the development of GD2 conditional models. The first model presents a loss of β-GC activity in all tissues, except epidermal tissue (Enquist et al., 2007). The second model presents a loss of β-GC activity restricted to neuronal and glial progenitors (Enquist et al., 2007). These animal models present neuronopathic symptoms such as abnormal gait, hyperextension of the neck, and seizures with microglial activation and neuronal cell loss (Enquist et al., 2007). Unlike *GBA1* KO mice, these conditional models have a longer life expectancy of 20–35 days, which provides an opportunity to study molecular pathways or treatments for more extended periods.

A new model of the chronic neuronopathic form was recently developed through the insertion of a Gba transgene regulated by a doxycycline system (Pewzner-Jung et al., 2021). The mice presented decreased β-GC activity levels in many brain areas and the liver, with GluCer accumulation and related motor and behavioral symptoms (Pewzner-Jung et al., 2021). This model resembles a GD3 phenotype with a longer survival period (10 months) and is proposed as an ideal model for studying the GD and PD relationship.

Since the link between GD and PD is an emerging topic, new GD models have been developed to uncover this relationship. A *GBA1* non-sense mutant in medaka fish presents a 50% reduction in β-GC activity associated with neuronal pathology, showing progressive cell loss (TH+ neurons), microglial activation, and α-syn accumulation (Uemura et al., 2015). Additionally, mutations in the *GBAb* ortholog in *D. melanogaster* led to decreased β-GC activity, substrate accumulation, lysosomal swelling, activation of the unfolded protein response (UPR), and inflammatory pathways (Cabasso et al., 2019). These studies show the development of potential models for GD research that can resemble neuropathology and improve life span(Table 2).

**Table 2:** Animal models designed for GD study.

**Model**	**Organism**	**Life span**	**β-GC activity**	**CNS accumulation**	**Gaucher cells**	**References**
*GBA1* knock-out	Mouse	24 h	<4%	Yes	Yes	Tybulewicz et al., 1992
RecNcil	Mouse	24 h	<9%	Yes	No	Liu et al., 1998
L444P/L444P	Mouse	24–48 h	20%	No	No	
L444P/UGCG	Mouse	1 year	15–20%	No	No	Mizukami et al., 2002
N370S/N370S	Mouse	24h	NA	NA	NA	Xu et al., 2003
V394L/V394L	Mouse	78 weeks	<27%	No	Yes	
V394L/null	Mouse	>42 weeks	<23%	No	Yes	
D409H/D409H	Mouse	68 weeks	<27%	No	NA	
D409H/null	Mouse	>42 weeks	<23%	No	Yes	
D409V/D409V	Mouse	66 weeks	<22%	No	NA	
D409V/null	Mouse	>42 weeks	<21%	No	Yes	
Mx1-Cre-LoxP	Mouse	Normal life span	<20%	No	Yes	Enquist et al., 2006
Tie2-Cre-LoxP	Mouse	NA	<50%	No	Yes	Sinclair et al., 2007
Mx1-Cre-LoxP	Mouse	NA	5%	No	Yes	Mistry et al., 2010
K14-and/lnl	Mouse	<20 days	<10%	Yes	Yes	Enquist et al., 2007
Nestin flox/flox	Mouse	<35 days	<10% Brain	Yes	NA	
*GBA1^−/−^; GBA1^*tg*^*	Mouse	10 months	30% Brain, 50% Liver	Yes	NA	Enquist et al., 2006
*GBA1^−/−^*	Fish	3 months	50%	Yes	Yes	Uemura et al., 2015
*GBA1bm*	Fly		Undetectable	Yes	NA	Cabasso et al., 2019

*NA, Not described*.

## Signaling pathways related to organelle dysfunction in nGD

The loss of β-GC activity and accumulation of GluCer trigger lysosomal dysfunction, impacting the function of other organelles due in part to alterations in specific signaling pathways. For instance, iPSC/GD-neuronal progenitors and IPCS/GD-neurons from GD2 patients show hyperactivity of the mammalian target of rapamycin complex 1 (mTORC1) kinase, which inhibits the function of the lysosomal master transcription factor TFEB (Brown et al., 2019). This transcription factor is responsible for inducing the expression of several lysosomal biogenesis and autophagy-related genes (Brown et al., 2019). The treatment of IPCS/GD-neurons with torin 1, a mTORC1 inhibitor, induces TFEB nuclear translocation, thus promoting lysosomal biogenesis, increasing LC3II/LAMP1 association, and decreasing p62 levels (Brown et al., 2019; Srikanth et al., 2021).

Furthermore, the necroptotic cell death pathway could be activated in nGD (Vitner et al., 2014; Yañez et al., 2021). In a genetic mouse model of GD2, neuronal cell death was not associated with increased apoptosis markers, but rather with elevated mRNA and protein levels of RIPK1 and RIPK3 – two kinases involved in necroptotic cell death (Vitner et al., 2014). Interestingly, RIPK3 knock-out mice exhibited decreased inflammatory signs in the CNS and improved motor symptoms and mouse lifespan (Vitner et al., 2014). Likewise, there was RIPK3 activation in different pharmacological and genetic nGD models, including patient fibroblasts (Yañez et al., 2021), CβE-treated neuronal lines, and nGD mice, which supported the key role of RIPK3 in nGD pathology (Yañez et al., 2021).

Interestingly, c-Abl tyrosine kinase is activated in different GD models. c-Abl signaling has been involved in the pathogenic mechanisms of several lysosomal storage disorders (LSDs). For instance, c-Abl is activated in Niemann–Pick C (NPC), a disease characterized by cholesterol accumulation in lysosomes. In NPC models, c-Abl signaling leads to increased p73 pro-apoptotic protein levels (Klein et al., 2011), amyloid precursor protein (APP) amyloidogenic processing (Yáñez et al., 2016), increases in histone deacetylase 2 (HDAC2) levels, repression of neuronal and synaptic genes, and retention of TFEB in the cytoplasm (Gonzalez-Zuñiga et al., 2014). Moreover, the c-Abl pharmacological inhibition in NPC models reduced cerebellar apoptosis and promoted autophagy and cholesterol clearance—changes associated with increased TFEB nuclear localization, TFEB target gene expression, lysosomal biogenesis, and exocytosis (Contreras et al., 2020). Recently, we found that the inhibition of c-Abl is also involved in alterations in the autophagy lysosomal pathway in Niemann–Pick A (NPA), a disease in which sphingomyelin accumulates in lysosomes (Marín et al., 2022). The inhibition of c-Abl improves autophagy lysosomal function, restoring autophagy flux and sphingomyelin accumulation in NPA models (Marín et al., 2022). These results show that alterations in c-Abl signaling are relevant in LSDs and suggest that it could also play an essential pathogenic role in GD.

Interestingly, in GD models, there is c-Abl activation and crosstalk between c-Abl and the RIPK1/RIPK3/MLKL pathway (Yañez et al., 2021). c-Abl interacts with RIPKP3, and its genetic inhibition decreases RIPKP3 levels. c-Abl mediates RIPK3 tyrosine phosphorylation in both GD neuronal model and fibroblasts from GD patients. These results suggest that c-Abl could play an important role in GD pathology by regulating RIPK3 signaling.

Thus, lysosome dysfunction and lysosome-altered signaling pathways are central to GD pathology. Nevertheless, the lysosome is a dynamic organelle. Its functions impact the function of other organelles through direct and indirect signaling pathways and membrane contact sites (MCSs) that dynamically regulate the morphology, fusion–fission events, distribution, and organelle function. Therefore, the disruption of lysosome integrity, such as β-GC deficiency and GluCer accumulation, will affect other organelles beyond just the lysosome.

## Dysregulation of organelles in nGD

### Lysosomal dysfunction: The central axis of nGD pathology

**Figure 1:**
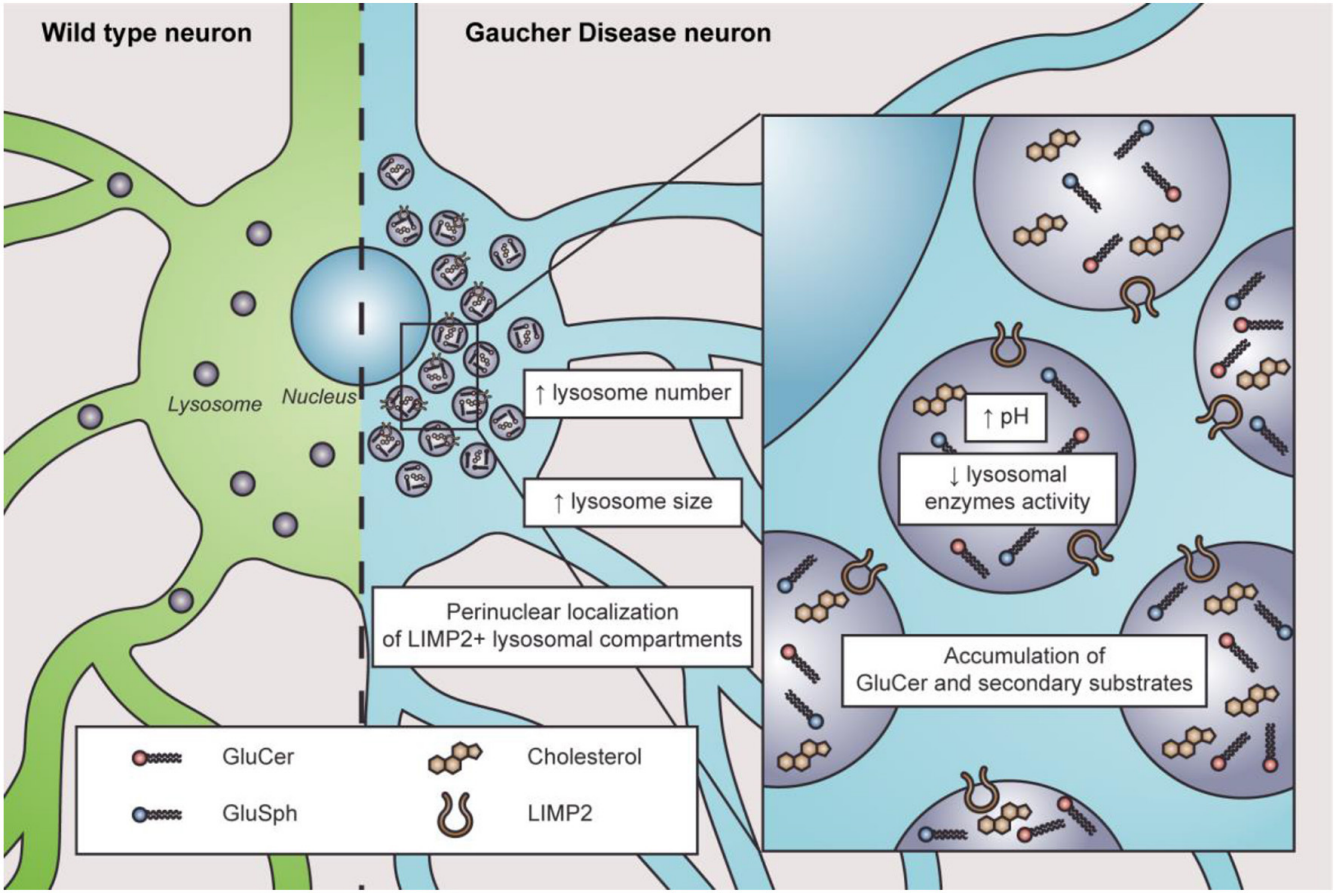
Characteristics of lysosome dysfunction in nGD. In physiological conditions, lysosomes are distributed throughout the cytosol in wild-type neurons (left in green). In nGD, lysosomes are increased in number and size with a perinuclear localization of LIMP2+ lysosomal compartments accompanied by elevated intraluminal pH and decreased lysosomal enzyme activity, along with the accumulation of GluCer and secondary substrates such as glucosylsphingosine (GluSph) and cholesterol (right in light blue).

The lysosome is the most affected organelle in GD since β-GC deficiency and GluCer accumulation alter the lysosome number, distribution, pH, enzymatic content, and activity (Figure 1). For instance, neuroblastoma cells with a *GBA1* mutation show an abnormal increase in acidic punctate structures positive to lysotracker staining; this could be associated with a higher number of acidic compartments (Schöndorf et al., 2014; García-Sanz et al., 2017). Likewise, iPSC-derived neurons from *GBA*1-associated PD patients showed an increased number and size of LAMP1-positive structures (Schöndorf et al., 2014; García-Sanz et al., 2017). One possible interpretation is that cells may respond to increasing lysosomal biogenesis to compensate for the lysosomal dysfunction induced by the loss of β-GC activity. Alternatively, the increased number of LAMP1-positive structures could indicate an accumulation of damaged lysosomes.

Altered lysosome distribution with preferentially perinuclear distribution is an early characteristic of nGD (Awad et al., 2015, 2017; Zigdon et al., 2017). The cause of altered lysosome distribution is unclear. However, cytoskeletal impairments are related to altered lysosome distribution since microtubule stabilization with paclitaxel decreases the percentage of altered lysosome distribution in neurons treated with CβE (Zigdon et al., 2017). Moreover, in a GD2 model, only LIMP2-positive lysosomes are found in the perinuclear region, observing no changes regarding LAMP1-positive lysosomes (Zigdon et al., 2017), which suggests that different lysosome subtypes might exist in nGD. Interestingly, LIMP2 is responsible for β-GC trafficking to the lysosomes (Reczek et al., 2007), and its downregulation is associated with reduced β-GC activity (Thomas et al., 2021). Additionally, lysosome localization depends on their maturation state since immature lysosomes are preferentially distributed in the cytoplasm periphery, while mature lysosomes are more abundant in the perinuclear region (Pu et al., 2016; Oyarzún et al., 2019). Lysosomal pH plays a pivotal role in lysosomal enzymatic activities. In this sense, cathepsins and acid sphingomyelinase present lower enzymatic activity in many cellular models of nGD (Tatti et al., 2012; García-Sanz et al., 2017; Panicker et al., 2018; Polinski et al., 2021). Indeed, a higher lysosomal pH from 5.5 to 6 was reported in astrocytes with *GBA1* mutations, RAW macrophages treated with CβE, and lymphoblasts derived from GD patients (Sillence, 2013). The increased pH in GD pathology is remarkable since the change in lysosomal pH in other LSDs is subtle (Sillence, 2013). Accordingly, defects in lysosome exocytosis were observed in osteoclasts and iPSCs from nGD patients, which was also related to lower membrane repair capacity (Panicker et al., 2018).

### The parallel alterations of the endoplasmic reticulum in nGD

**Figure 2:**
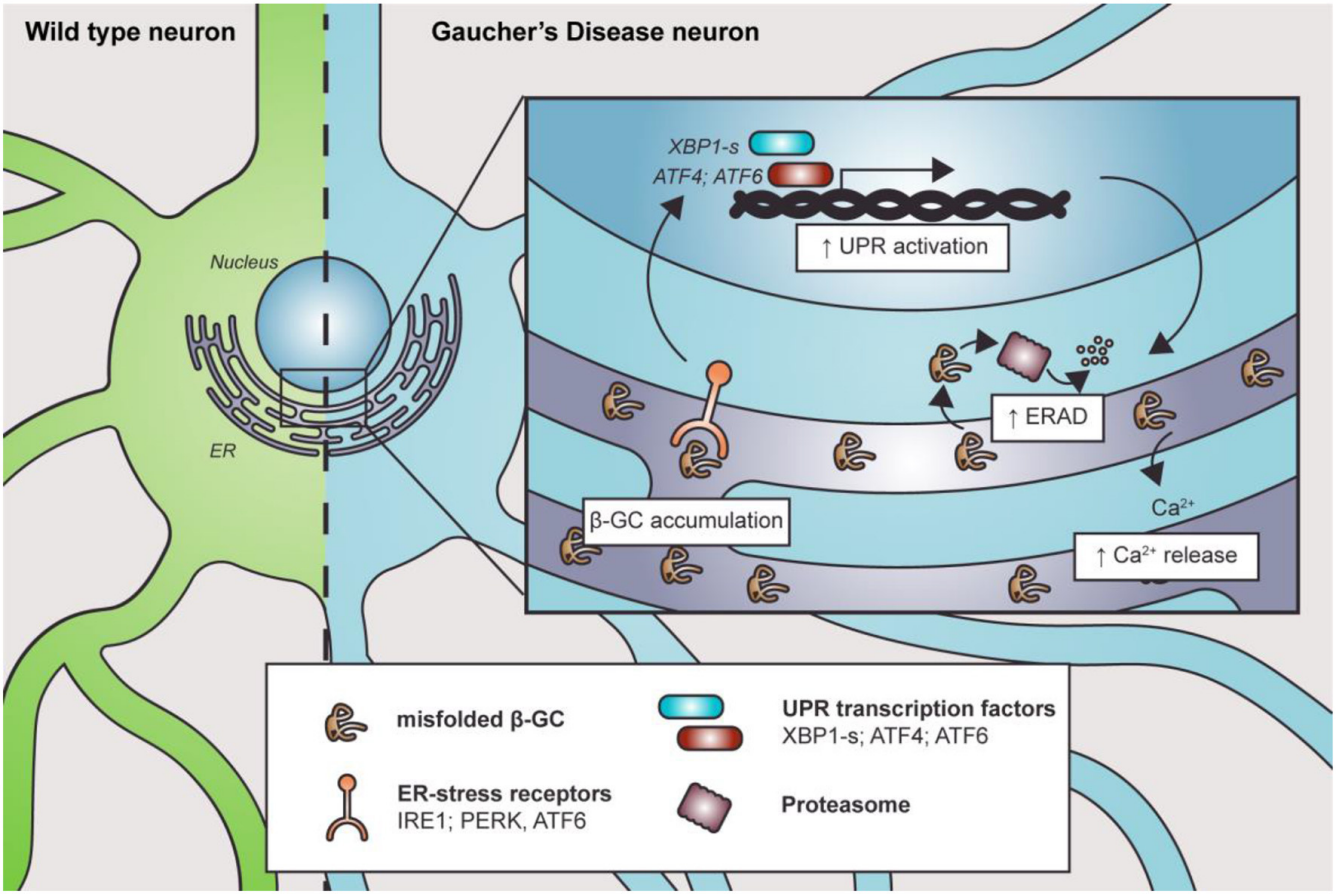
Misfolded β-GC mediates ER stress. Mutations in *GBA1* lead to β-GC misfolding, promoting the activation of ER stress receptors and the UPR. This, in turn, induces degradation of the misfolded enzyme through ERAD. Along with this, Ca^2+^ homeostasis is also altered, contributing to cell and organelle damage.

The endoplasmic reticulum is the first station where β-GC transits and folds. Therefore, it is intuitive to think that the mutations could cause changes in its tertiary structure and folding, impacting the ER state. Endoplasmic reticulum stress, ERAD overactivation, and UPR response were reported in several nGD models (Figure 2).

The expression of human mutant β-GC was linked with ER defects and activation of UPR signaling in *D. melanogaster* (Maor et al., 2013a,b). Furthermore, the mutant forms of β-GC colocalize with ER–protein calnexin, a chaperone that participates in the pro-folding pathway and degradation of misfolded proteins, in fibroblasts from nGD patients (Maor et al., 2013a,b). Interestingly, the level of β-GC/calnexin colocalization is related to disease severity (Maor et al., 2013a,b). Similarly, the ubiquitin E3 ligase ITCH, known as atrophin-1-interacting protein 4, binds to mutant, but not to the wild-type, β-GC to mediate its ERAD degradation, another parameter that has been related to disease severity (Maor et al., 2013a,b).

In the *D. melanogaster* nGD model, the expression of human mutant β-GC leads to neurodevelopmental eye defects and UPR signaling activation (Suzuki et al., 2013). Exacerbated or prolonged misfolded protein accumulation could trigger ER stress and activation of the UPR (Suzuki et al., 2013). Accordingly, treatment with ambroxol, a pharmacological chaperone that promotes β-GC folding and trafficking, reverts ER stress and neurodevelopmental defects (Suzuki et al., 2013). Also, GD patients' fibroblasts and iPSCs carrying a heterozygous *GBA1* mutation showed increased spliced xbp1 mRNA levels, upregulation of ER dBiP and CHOP proteins, and increased eIF2α phosphorylation (Wei et al., 2008; Maor et al., 2013b; Fernandes et al., 2016). The increase in dBip and xbp1 mRNA levels suggests the activation of the IRE1a branch of the UPR, probably due to ER stress. Additionally, the elevated levels of Xbp1 spliced mRNA could explain the upregulation of CHOP, a target of the XBP1s transcription factor. This branch could activate apoptosis in response to stress. The IRE1a/XBP1 branch of the UPR correlates with the expression of other proteins such as ERAD and ERAD-enhancing α-mannosidase-like proteins (EDEMs). Moreover, GD1 and GD2 fibroblasts showed increased Grp78/Bip, Grp94, ATF6, and SOD2 protein levels, which are important ER chaperones. Also, these cells are more susceptible to brefeldin-mediated apoptosis (Wei et al., 2008). The increased misfolded protein response is probably due to the accumulation of β-GC, which releases Grp78/Bip from the UPR receptor and activates different pathways, including the ATF6 UPR branch (Wei et al., 2008). One of the ATF6 target genes is Xbp1, which amplifies the mis-/unfolded protein response. Another ATF6 target gene is CHOP, suggesting that GD fibroblasts are more prone to cell death under stressful conditions (Wei et al., 2008).

Intriguingly, it seems that β-GC accumulation in the ER, and not lysosomal substrate accumulation, is the cause of UPR activation since β-GC inhibition with CβE does not lead to UPR activation in healthy fibroblasts (Maor et al., 2013b). UPR activation was also corroborated in *D. melanogaster* GD models (Maor et al., 2013b; Sanchez-Martinez et al., 2016). In this regard, the treatment of flies with an isofagomine, a chaperon that promotes protein folding, decreased Xbp-1 protein levels and improved the decline in climbing caused by the expression of mutant β-GC (Sanchez-Martinez et al., 2016). Moreover, Mu et al. showed promising results using the celastrol chaperone in skin GD fibroblasts carrying the L444P mutation, which increases β-GC activity, folding, and trafficking to the lysosome and avoids activation of the ERAD response. These effects are partially mediated by UPR activation since inhibition of ATF6, IRE1, and PERK blocks the effects of celastrol (Mu et al., 2008). These results open the possibility that at least some mutations found in the *GBA1* gene could be treated by chemical chaperones or by ERAD enhancers to diminish the levels of misfolded β-GC.

ER stress could also be mediated by dysregulation of calcium (Ca^2+^) ER homeostasis. The first evidence of the relationship between β-GC deficit and Ca^2+^ alterations was described by Korkotian et al. (1999). These authors showed that β-GC inhibition with CβE in hippocampal neurons leads to a three-fold increase in ER volume, observing tubular membrane structures, accompanied by increased ryanodine receptor (RyR) levels and Ca^2+^ release after glutamate or caffeine treatment (Korkotian et al., 1999). Furthermore, CβE-treated cells were more sensitive to glutamate-induced neuronal cell death, which was blocked by pre-incubating with ryanodine. This suggests that increased Ca^2+^ release from the ER could contribute to neuronal death in neuronopathic forms of GD (Korkotian et al., 1999).

Pelled et al. showed that microsomes from the brains of GD2 patients had higher Ca^2+^ release than microsomes from GD1 patients, GD3 patients, and healthy subjects. Additionally, Ca^2+^ positively correlated with the level of GluCer accumulation in the same microsomes (Pelled et al., 2005). Furthermore, treatment with thapsigargin, an inhibitor of the ER-Ca^2+^ ATPase, induces an elevated release of Ca^2+^ in fibroblasts from GD1 patients and PD patients carrying *GBA1* mutations (Kilpatrick et al., 2016). These facts support the idea that the accumulation of the misfolded enzyme in the ER could contribute to toxic GluCer accumulation in the lysosome (Kilpatrick et al., 2016). In conclusion, the ER is affected by nGD and contributes to cellular alterations. This opens the question of whether lysosomes are the central organelle affected by Gaucher disease or whether there is a parallel dysfunction in the ER due to the misfolded enzyme.

### Autophagosome: Early and late failures of autophagy in nGD

Macroautophagy (hereafter referred to as autophagy) is necessary for proper cell function. There are two main types of autophagy: bulk autophagy, where the cytoplasmic material is recycled, and selective autophagy, where specific organelles or materials, such as aggregated proteins, are sequestered by the autophagy machinery to be degraded after its fusion with the lysosome (Ravikumar et al., 2009). Several studies have demonstrated increased autophagy markers, such as the lipidated forms of LC3, LC3-II, and SQSTM1/p62 in nGD models (Tatti et al., 2012; Schöndorf et al., 2014; Awad et al., 2015; Bae et al., 2015; Du et al., 2015; Aflaki et al., 2016; García-Sanz et al., 2017; Brown et al., 2019; Li et al., 2019). This increase in autophagy protein levels seems to be related to a defect in autophagy flux, rather than a consequence of increased autophagosome biogenesis. For instance, it has been proposed that GluCer accumulation leads to a direct decrease in autophagosome/lysosome fusion (Awad et al., 2015; Du et al., 2015) and, consequently, defects in autophagosome clearance and protein accumulation (Awad et al., 2015, 2017; Du et al., 2015). These defects in lysosomal degradation capacity and autophagic flux have also been observed in pharmacological and genetic nGD animal models. For example, mice treated with CβE present increased levels of SQSTM1/p62 in the substantia nigra and cortex, two main areas affected in GD (Rocha et al., 2015). Likewise, increased levels of these proteins were observed in mice with a *GBA1* L444P heterozygous mutation, supporting the idea that deficient β-GC activity leads to autophagic defects (Li et al., 2019). Mice with mutations on *GBA1* and *Sap C*—which encode the lysosomal β-GC activator saposin C—also present increased SQSTM1/p62 levels in the brain stem, basal ganglia, and thalamus. Similar results were observed in new models of *D. melanogaster*, which contain altered Ref(2)P and ATG8—homologs of p62/SQSTM1 and LC3, respectively (Davis et al., 2016).

In addition to substrate accumulation, protein degradation through autophagy and chaperone-mediated autophagy (CMA) is impaired in nGD (Sun and Grabowski, 2010). In line with this, α-syn is a protein degraded by both types of autophagy (Kuo et al., 2022). Some authors have not observed accumulation of its toxic status in the brains of infants with GD2, although altered plasma α-syn levels were reported (Berger-Sieczkowski et al., 2016). Additionally, other authors observed inclusions of α-syn in the brains of GD patients with parkinsonism (Wong et al., 2004), raising the question of whether α-syn accumulation is directly linked to β-GC activity in GD or is it a result of the parkinsonism present in some GD patients.

Several studies show that a reduction in β-GC stabilizes α-syn, promoting its aggregation (Manning-Bog et al., 2009; Mazzulli et al., 2011; Taguchi et al., 2017). However, the mechanisms by which β-GC deficiency promotes α-syn aggregation are controversial. Some authors suggest that GluCer accumulation itself could induce the formation of soluble oligomeric intermediates, thus influencing the amyloid formation of purified α-syn (Mazzulli et al., 2011). At the same time, others suggest that insufficient or mutated β-GC could promote the formation of these aggregates. Supporting the latter idea, neurons treated with CβE neither increase insoluble monomeric α-syn levels nor its phosphorylation status (Gegg and Schapira, 2016). Correctly folded β-GC can interact with α-syn in a way that promotes its degradation (Yap et al., 2015), while the GD-related mutation N370S could weaken this interaction, increasing the probability of α-syn aggregation (Yap et al., 2013). This toxic aggregation contributes to autophagy dysfunction, resulting in a bidirectional loop since toxic α-syn can block β-GC trafficking to lysosomes. By contrast, the autophagy machinery, including the ATG8-independent CMA, cannot degrade these toxic aggregates (Mazzulli et al., 2011; Kuo et al., 2022).

Along with lysosomal-related dysfunction in autophagy, initial autophagy steps were also altered. Accordingly, the hippocampus of L444P heterozygous mice showed elevated levels of LC3-II and SQSTM1/p62 compared with wild-type (WT) animals (de la Mata et al., 2015). Moreover, the treatment of *GBA1* mutant neurons with the autophagy inducer rapamycin and autophagy flux blockers, leupeptin, and pepstatin diminishes the increase in autophagic vacuoles compared to WT neurons, indicating that both early and late autophagies are altered in GD (Li et al., 2019).

In another nGD model, LC3-II levels were reduced in *GBA1*^−/−^ cells compared to WT cells. This observation could be due to impaired LC3 conjugation with phosphatidylethanolamine since ATG5/12 levels were also reduced. Consistent with this, astrocyte *knock-out* in *GBA1* induces a diffuse cytoplasmic distribution of LC3 and impairs the formation of autophagic structures (Osellame et al., 2013). These differences in autophagy marker levels and LC3 distribution seem to be related to the extent of β-GC deficiency since *GBA1*^+/−^ astrocytes can form autophagosomes (Osellame et al., 2013). Accordingly, no accumulation of β-GC substrates was detected in GBA^+/−^ mouse models or the brains of PD patients with heterozygous *GBA1* mutations (Sardi et al., 2011; Farfel-Becker et al., 2014; Gegg and Schapira, 2016).

### Mitochondrial damage as a prominent feature in nGD

Altered mitochondrial homeostasis and its consequences are widely described in other neurodegenerative diseases and LSDs (Osellame and Duchen, 2014; Stepien et al., 2020). In nGD, some studies show altered mitochondrial morphology, membrane potential, and energetic metabolism. Specifically, decreased mitochondrial membrane potential (ΔΨm), fragmented mitochondria, reduced ATP production levels, and increased ROS production have been observed in different nGD models (Cleeter et al., 2013; Osellame et al., 2013; Dasgupta et al., 2015; de la Mata et al., 2015; Yun et al., 2018; Ivanova et al., 2019; Li et al., 2019; Morén et al., 2019). These effects were previously noticed in later treatment days with CβE in SH-SY5Y cells (Cleeter et al., 2013). Furthermore, *GBA1*^−/−^ neurons and astrocytes showed increased mitochondrial mass due to blocked autophagy flux. Interestingly, the incubation of cells with MITOQ10, a mitochondrial antioxidant, does not change LC3 II protein levels, which are decreased in this model. This suggests that mitochondrial alterations are secondary effects of organelle dysfunction and defective degradation of these mitochondria *via* autophagy (Osellame et al., 2013). Additionally, dysfunctional mitochondria in *GBA1*^−/−^ neurons and astrocytes could not recruit Parkin, a ubiquitin E3 ligase essential for marking mitochondria for autophagy degradation, and these cells were unable to form autophagic vesicles (Osellame et al., 2013).

By contrast, GD fibroblasts harboring L444P/L444P mutation presented elevated LC3 II, a block in the autophagic flux, and accumulated autophagic vesicles containing depolarized mitochondria. This evidence suggests that mitophagy activation and impaired autophagic flux are present in this GD model (de la Mata et al., 2015). Furthermore, these differences in altered mitophagy regulation seem to be related to the specific Gaucher model since *GBA1*^+/−^ astrocytes, but not *GBA1*^−/−^, can form autophagic vacuoles that could sequester dysfunctional mitochondria (Osellame et al., 2013).

Thus far, it is unknown whether mitochondrial dysfunction is a direct consequence of lysosomal dysfunction or if other cellular events contribute. Recently, Kim et al. showed that β-GC mutations in dopaminergic iPSC-derived neurons harboring heterozygous *GBA1* mutations led to prolonged mitochondria–lysosome (M-L) contact sites compared to isogenic controls. This correlated with decreased mitochondrial density in axons. Increased proteasomal degradation of TBC1C15—a Rab7 GAP, which facilitates GTP hydrolysis and mitochondria–lysosome untethering—could cause these prolonged contacts (Kim et al., 2021). Interestingly, this altered mitochondria–lysosome dynamic was reproduced in the pharmacologic nGD model, and restoring β-GC function retrieved this long mitochondria–lysosome contact phenotype. Additionally, inhibition of other lysosomal enzymes does not disrupt mitochondria–lysosome contacts in neurons, suggesting that β-GC and GluCer exert a pivotal role in mitochondria–lysosome dynamics (Kim et al., 2021).

A greater colocalization coefficient of β-amyloid precursor protein (APP) and α-syn inclusions with the mitochondrial proteins TOM40 and COX IV were observed in cortical neurons of an nGD mouse model (Xu et al., 2014). By contrast, a small portion of these proteins colocalized with autophagosome markers LC3 II and p62 or with the lysosome (LAMP2) (Xu et al., 2014). These results suggest preferential clustering of APP/α-syn in mitochondrial compartments in cortical GD neurons, showing electron-dense and enlarged mitochondria with functional defects (Xu et al., 2014). The altered influx of APP/α-syn could block mitochondrial protein import channels, leading to the arrest of essential proteins for mitochondrial function (Devi et al., 2006). The chronic neuroinflammatory environment and decreased antioxidant compounds in GD could play a pivotal role in mitochondrial dysfunction.

### Emerging evidence of organelle dysfunction in GD pathology

Although the lysosome, ER, and mitochondria are the prominent organelles explored in nGD pathology, other organelles could also play a pivotal role. In fibroblasts harboring the N370S *GBA1* mutation, the Golgi apparatus (GA) alterations are striking, showing a fragmented structure, dispersed into small elements. Compared to control cells, heterozygous *GBA1* mutants have shorter and smaller GA cisternae (García-Sanz et al., 2017). Alteration of the Golgi apparatus structure could have functional consequences. For instance, inhibition of β-GC by CβE alters lactosylceramide sorting from the GA to the lysosome, suggesting that loss of β-GC activity impacts GA-related traffic, which could be related to alterations in its structure or functional components (Sillence et al., 2002).

Likewise, exosomes have gained increased attention since they participate in cellular communication and signaling in a bidirectional way in health and disease. Interestingly, plasma from GD patients presents large and multilayered exosomes with various morphologies compared to healthy controls (Tatiana et al., 2020). Some of these exosomes were electron-dense and had a higher fluorescence intensity of exosome markers CD9 and CD81 than controls (Tatiana et al., 2020). In this regard, Papadopoulos et al. showed that β-GC inhibition increases the number of brain exosomes and α-syn-associated exosomes in an overexpressing α-syn mouse model treated with CβE. Although further studies are required to establish the exosome role in GD pathology, they may have a relevant role as a garbage transporter for clearing or transporting signal molecules that can contribute to or decrease the pathological environment (Papadopoulos et al., 2018).

## Conclusion

**Figure 3:**
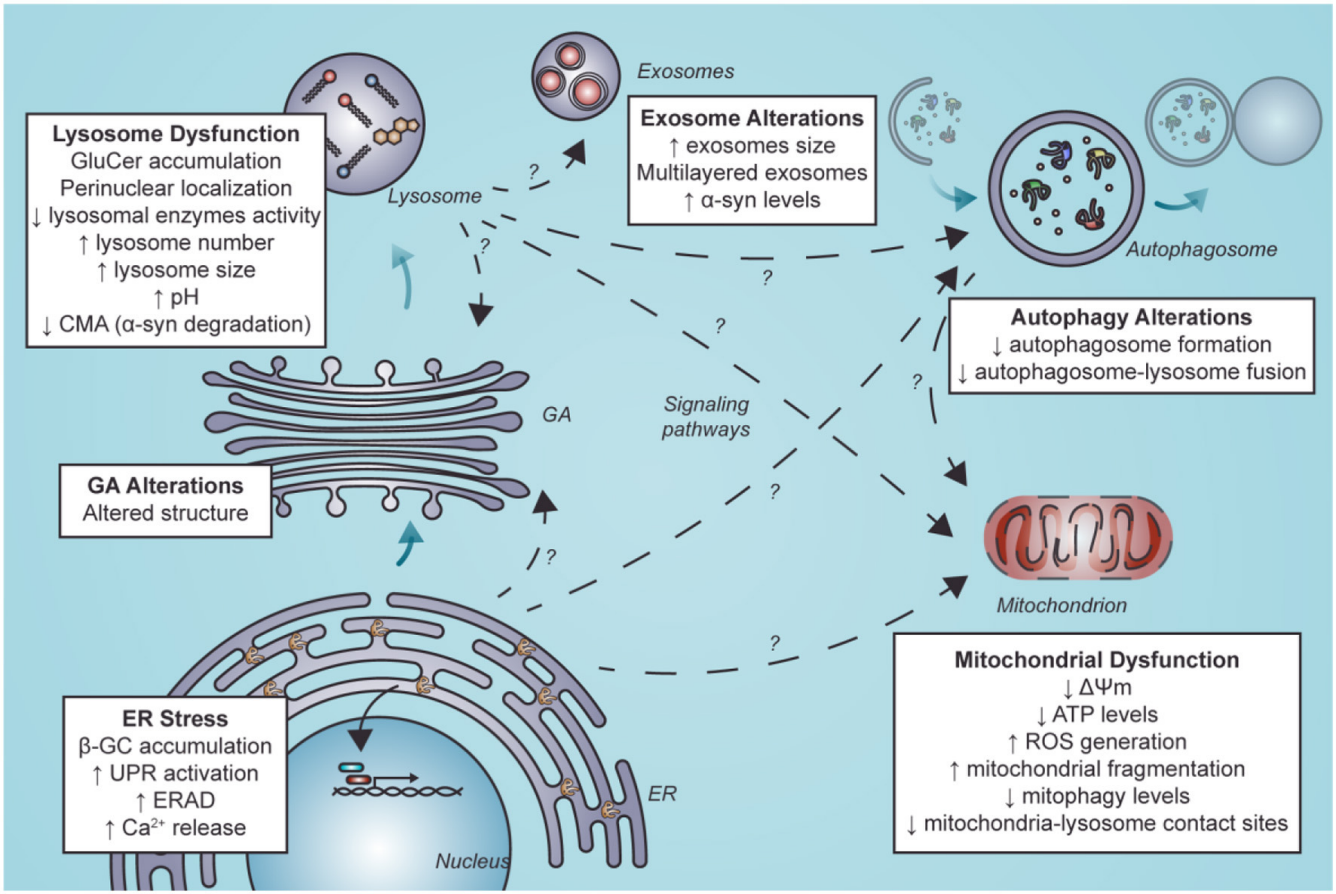
Secondary organelle dysfunction in nGD following ER stress and lysosome dysfunction. Lysosome dysfunction is characterized by GluCer accumulation, perinuclear localization of LIMP2-positive structures, diminished lysosomal enzymes activity, elevated lysosome number and size, alkalinization of lysosomes and decreased levels of CMA. Furthermore, GluCer accumulation and ER stress mediated by β-GC misfolding can alter other organelles. These alterations could be mediated by direct interaction with other organelles. They could also result from indirect interactions between lysosomes and other organelles by activating signaling pathways or alterations in these organelles such as exosomes, autophagosomes in autophagy, mitochondria, and GA. Alterations in autophagy and mitochondria have been well described. Still, little is known about the mechanisms that lead to these alterations and less about alterations in other organelles such as exosomes and GA (segmented arrows with a question mark). GluCer, glucosylceramide; CMA, chaperone-mediated autophagy; ER, endoplasmic reticulum; β-GC, β-glucocerebrosidase; GA, Golgi apparatus; ERAD, endoplasmic reticulum-associated degradation; ROS, reactive oxygen species.

This review aimed to evaluate the functional consequences of lysosomal dysfunction on other organelles, such as the ER and mitochondria (Figure 3).

nGD is an inherited recessive autosomal metabolic defect due to deficiency of the lysosomal enzyme β-GC that induces neurodegeneration. Decreased catalytic activity and instability of β-GC lead to the accumulation of glucosylceramide, glucosylsphingosine, and cholesterol in the lysosomes of macrophage cells and visceral organs, predominantly in the liver, spleen, and bone marrow.

Dysfunction of the autophagy–lysosomal pathway represents a key pathogenic event in *GBA1*-associated neurodegeneration. The autophagy–lysosomal pathway maintains cellular homeostasis by clearing protein aggregates and damaged organelles, a process critical for neuronal survival. However, the pathological consequences are not only due to the intrinsic β-GC enzymatic dysfunction but also the consequence of abnormalities occurring during its transport and delivery to the lysosome. Thus, β-GC misfolding during its passage through the ER can lead to premature degradation by the proteasome, activating ER stress receptors and the UPR. Along with this, Ca^2+^ homeostasis is also altered, contributing to cell and ER damage. Furthermore, mutant forms of β-GC colocalize with calnexin (Ron and Horowitz, 2005), and there is a significant correlation between disease severity and the level of β-CG calnexin interaction (Ron and Horowitz, 2005). In line with this, the reduction of the β-CG degradation rate promotes its delivery to the pro-folding calnexin pathway and increases enzymatic activity in Gaucher disease fibroblasts (Tan et al., 2014).

On the other hand, mitochondrial dysfunction is emerging as a significant contributor to the pathophysiology of lysosomal storage disorders. Lysosomes are essential for autophagy and autophagic clearance of dysfunctional mitochondria and represent an essential element of mitochondrial quality control. We suggest that the compromised mitochondrial function in these cells may increase the risk of neurodegeneration in neurons that are already vulnerable because of their normal physiological activity.

Although lysosomal dysfunction is key to the loss of cell homeostasis in nGD, other organelles could be affected as a direct or indirect consequence of lysosome perturbation. In this regard, modulation of specific mitochondrial and endoplasmic reticulum pathways could be a new therapeutic frontier in GD management. We propose that other cell components such as GA and exosomes could play a role yet to be uncovered in nGD pathology.

## Author contributions

NA, AÁ, MY, and SZ: writing—original draft preparation. NA, CL, VC, PB, AÁ, MY, and SZ: conceptualization and writing—review and editing. AÁ, MY, and SZ: funding acquisition. All authors have read and agreed to the published version of the manuscript.

## Funding

This work was supported by Agencia Nacional de Investigación y Desarrollo (ANID), FONDECYT [Grants Nos. Q221201668 (AÁ), 11200592 (MY), and 1190334 (SZ)], FONDEF ID21I10347, Millennium Science Initiative Program ICN 09_016/ICN 2021_045, Millennium Institute on Immunology and Immunotherapy, and Centro de Envejecimiento y Regeneración CARE-Chile-UC Center under grant number AFB170005 (AÁ and PB).

## Conflict of interest

The authors declare that the research was conducted in the absence of any commercial or financial relationships that could be construed as a potential conflict of interest.

## Publisher's note

All claims expressed in this article are solely those of the authors and do not necessarily represent those of their affiliated organizations, or those of the publisher, the editors and the reviewers. Any product that may be evaluated in this article, or claim that may be made by its manufacturer, is not guaranteed or endorsed by the publisher.
